# Comment to the Article “Prevention of B Thalassemia in Northern Israel - A Cost-Benefit Analysis” by Koren et al.^1^

**DOI:** 10.4084/MJHID.2014.021

**Published:** 2014-03-09

**Authors:** Antonio Amato

**Affiliations:** Centro Studi Microcitemie Roma

Thalassemias and hemoglobinopathies must be considered the most common genetic blood disease in the world: in fact, according to the World Health Organization (WHO), 7% of the global population is a carrier for a hemoglobin disorders.^2^ For this reason, these diseases represent a global public health problem; that is worldwide common; in addition, current widespread migrations have exacerbated the situation, by diffusing these diseases from their native areas to other countries in which they are becoming endemic. The issue of migration flows has “reshuffled the cards”, not only in those countries in which hemoglobinopathies and thalassemias were, until now, nearly unknown, but also in countries whose structures were already well organized to support the usual load of patients.^3^ The massive dimension of migration events is about to clog the various National Health Services, not only for mere economic reasons. As a result of this changed situation, have emerged several needs related to the care of the patients, including overload transfusion requirements, bone marrow transplantation requests, the onset of collateral diseases (hypothyroidism, diabetes mellitus, osteoporosis…) and others; all these issues are in the limelight today because of the increasing number of immigrant patients. Due to the rising number of affected individuals, several International Organizations, that deal with public health, have given indication to their relative National Health Systems to take control of the organization of prevention programs, that are aimed at both precocious detection of carriers and treatment of patients.^4^

Although today we are looking for novel solutions using established procedures (as allogenic bone marrow transplantation) or testing alternative therapeutic approaches (as pharmacological induction of fetal hemoglobin synthesis or gene therapy strategies), such are the issues that prevention still remains the solution for the main part of the problems. In the mid 70s, prenatal diagnosis was introduced in Italy as a new form of prevention.^5^

In exchange for the abortion of the affected fetuses, the possibility to have healthy children to thalassemia carriers couples has been offered todays.

The obtained results and the increasingly wide consent might have given the impression that the problem of the thalassemia prevention was solved: it appeared to be sufficient to examine young couples and to recur to prenatal diagnosis in those cases in which both members were carriers of thalassemia. But, over the years, after this initial approach, more comprehensive prevention strategy, based on the identification of carriers well in advance of the procreation, has been established.

In non-endemic countries, newborn screening and earlier prenatal diagnosis carried out on chorionic villi have been implemented, but this is not enough.^6^ In respect of those people who deplore abortion – for ethical reasons or religious choices –preconception prevention programs need to be implemented. These programs are based on both early information and diagnosis of carriers: in fact, the more the information and diagnosis are precocious, the more the choices are thoughtful and aware. Contrary to the case of early information, the interventions that can be undertaken after conception prevent the couples from choosing a number of alternative options. Having had a precocious information, the carriers can choose of not to have a carrier partner, of not to have children, to adopt a child, to use heterologous fertilization (donor gametes), to use pre-implantation diagnosis and not to choose only prenatal diagnosis as the unique alternative to the acceptance of risk.

The prevention program carried out for many years by the Centro Studi Microcitemie of Rome (ANMI Onlus - CSMR) consists in school screening offered to children in the last year of middle school.^7^ In addition, being economically very advantageous (about € 6.00/test), this proposal results to be the most ethically acceptable. In this case, the information pertains and involves not only the young people who are directly involved, but also and especially their families, resulting in the final analysis as a valuable tool for education and health care for the entire population.^7^ Of course, the prevention program, implemented in Latium in preventing Thalassemia Major, can also be performed in other genetic diseases, such as Tay Sachs disease or Cystic Fibrosis. However, a similar screening programs of these hereditary diseases has been developed in Jewish community schools in Australia, and include mandatory on-site education followed by voluntary on-site genetic carrier testing.^9^

The precise evaluation of health costs presented by Ariel Koren and coauthors^1^ in the paper titled “Prevention of β-thalassemia in Northern Israel–A cost-benefit analysis” is very appreciable, but the activity of sanitary prevention, usable by a wide variety of people who are different for culture, religion and ethical values, can not be limited by mere economic evaluation. In particular, the early information given to healthy carriers must be considered: the costs of prevention programs offered at school age are fully justified if to these young thalassemia carriers is given the opportunity of freely and timely choosing among different preventive options, not limiting them to the only choice of therapeutic abortion, that remains a painful, emotionally heavy and drastic solution.
